# Advancing migration and health research by examining return migration

**DOI:** 10.1136/jech-2023-220670

**Published:** 2024-01-05

**Authors:** Pilar Serrano-Gallardo, Rosa Mas-Giralt, Simone Castellani, Sol P Juarez

**Affiliations:** 1 Nursing Department, Universidad Autonoma de Madrid, Madrid, Spain; 2 Instituto de Investigación Sanitaria Puerta de Hierro Segovia de Arana (IDIPHISA), Madrid, Spain; 3 Research Institute for Higher Education and Science (INAECU), Madrid, Spain; 4 Lifelong Learning Centre, School of Geography, University of Leeds, Leeds, UK; 5 Department of General Economics, Faculty of Social and Communication Sciences, Universidad de Cadiz, Cadiz, Spain; 6 Department of Public Health Sciences, Stockholm University, Stockholm, Sweden; 7 Centre for Health Equity Studies, Stockholm, Sweden; 8 Karolinska Institute, Stockholm, Sweden

**Keywords:** HUMAN MIGRATION, Health inequalities, HEALTHCARE DISPARITIES, HEALTH POLICY

## Abstract

This essay offers an analysis of research on return migration and health by adopting the social determinants of health (SDH) framework proposed by the WHO. Specifically, we argue that the SDH are implicated in the decision to migrate, stay or return, which in itself also contributes to social health inequities. Most theoretical frameworks developed to study migration have predominantly considered primary migration movements. The lack of a fluid consideration of the migration phenomenon has a direct impact on our understanding of the relationship between migration and health. In this essay, we, first, address the challenges of defining and studying return and its implications for health research. Second, we propose to use the WHO’s SDH framework to understand how social factors shape migrants’ health, influence the decision to return and can contribute to health inequalities. The conceptual approach developed in this paper can help design future studies on the health of return migrants, fostering interdisciplinary collaborations to investigate how social factors are embodied, giving rise to health inequities in society that are intricately linked to the migration experience.

## Introduction

Most empirical studies and theoretical frameworks developed to study migration and health have considered exclusively or predominantly primary migration movements.^1^ Even though the International Organization for Migration (IOM)^2^ distinguishes between more than 20 forms of migration, migratory movements often continue to be conceptualised as a one-way process from a given origin to a given destination.

Return migration is the second most common migration trajectory. It is estimated that during the first 5 years, between 20% and 50% of migrants leave their destination, either to return to their country of origin or to move to another country.^3^ Dumont and Spielvogel^3^ show that return migration is more common among the youngest and the oldest migrants, as well as those with the highest and lowest educational levels. Additionally, these authors highlight that return migration occurs primarily between countries with similar levels of economic development.^3^ Chen *et al* 4 found that the highest percentage of return migration takes place in Latin America and the Caribbean (55.9%), and the lowest in Europe and North America (28.2%). Despite the significance of these figures, the health of returnees has generally been overlooked.

This paper provides a conceptual approach to research related to international return migration and health. We begin by addressing the challenges in defining and studying return and its implications for health research. Following this, we propose using the WHO′s social determinants of health (SDH) framework to understand how social factors shape immigrant health, influence the decision to return and contribute to health inequities. This work is part of the European project RETORNO. A realist synthesis has served as the basis for the proposal of this conceptual approach.

### Challenges of defining and identifying return migration and its implications for health research

**Figure 1:**
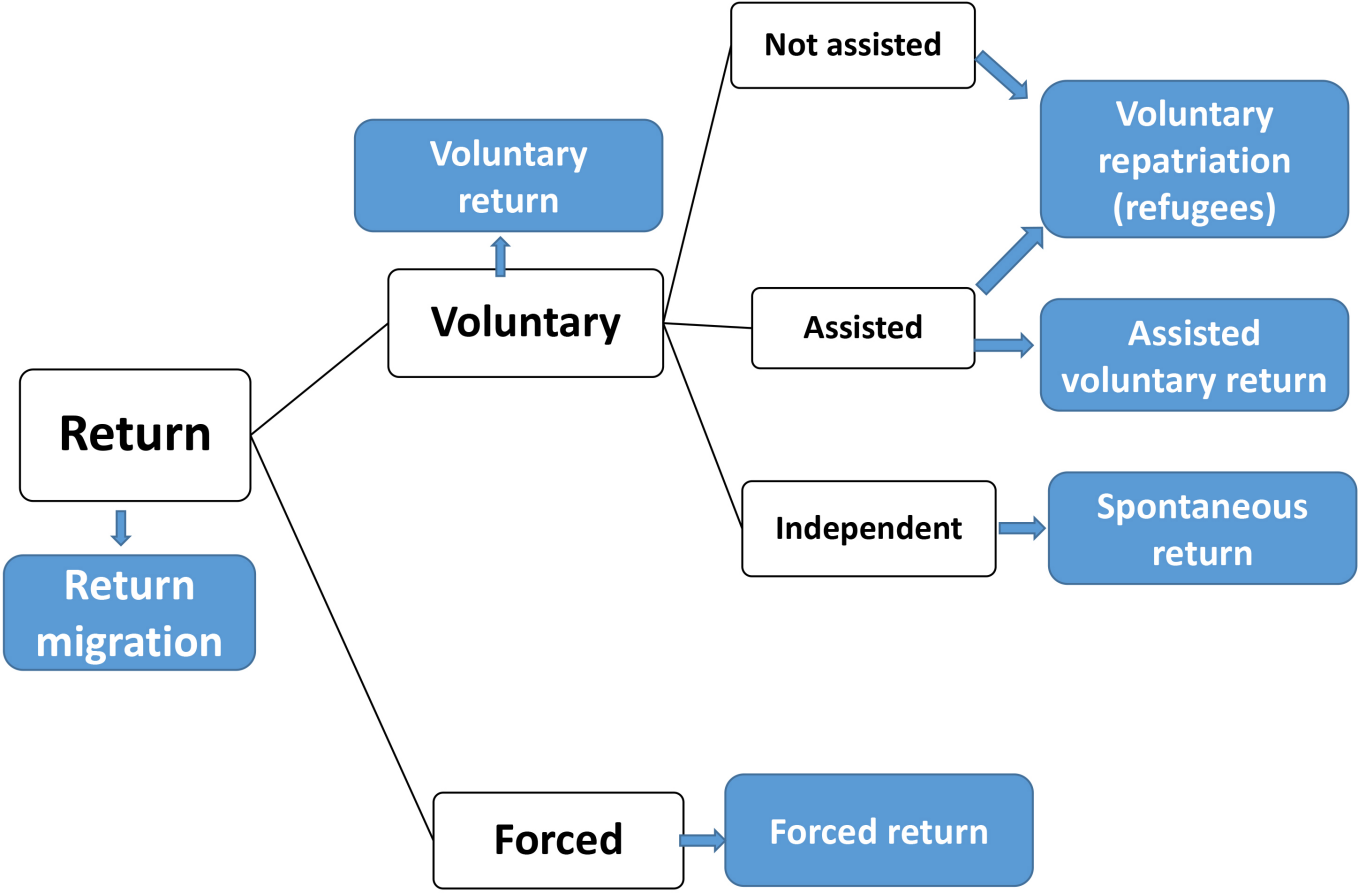
Types of return migration source: International Organisation for Migration.^2^

The IOM defines return migration as ‘the movement of persons returning to their country of origin after having moved away from their place of habitual residence and crossed an international border’.^2^ Return can be spontaneous or forced. Spontaneous return refers to voluntary and independent moves, generally without the support of states or other international or national assistance. Voluntary moves can also be assisted when there is administrative, logistical or financial support to migrants who cannot or do not want to stay in the receiving or transit country and who decide to return to their country of origin. It is also relevant to distinguish between assisted voluntary return and voluntary repatriation; the latter of which is linked to the refugee population and can be organised by institutions or by the refugees’ own means. In contrast to these spontaneous and voluntary types, there is forced return, which occurs against the will of the migrant and is generally carried out based on an administrative or judicial act or decision^2^ (figure 1).

The paucity of research on the health of returned migrants can be partially explained by the difficulties to identify those individuals who left a receiving country. While return migration is the movement of people returning to their country of origin,^2^ the operationalisation of this concept is challenging. For instance, to be considered a returnee, it is necessary to prove that a person has been resident abroad. Many migrants do not register themselves at the consulates of their country of origin due to a lack of awareness of the benefits of this registration, and/or to the inconvenience and expenses involved (eg, travel to another city). To capture the variability and dynamism of contemporary types of migration, the concept of ‘liquid migration’ has been proposed.^5^ This highlights the discrepancies between officially documented migration and unregistered flows, especially of regular and irregular labour migration; where circular, seasonal, non-linear movements are common.^5 6^

Moreover, it is particularly difficult to conceptualise and measure return in certain geopolitical contexts. This is the case, for example, of intra-European citizens because open-ended mobility is not adequately captured within migration processes.^7^ Thus, complex situations are often concealed within the definition of return migration (eg, repeated or secondary migration; temporary or permanent moves).^3^

Beyond statistical considerations, researchers from many different disciplines have long challenged the concept of ‘return migration’ as it implies that ‘home’ constitutes the end of the migration cycle.^8–10^ However, many trajectories are no longer linear, and mostly what exists are return visits, temporary returns and re-emigration; return is never a return to the starting point as it may be experienced as a new departure; the sense of belonging—that is, the ‘dynamic emotional attachment that relates people to the material and social worlds that they inhabit and experience’^11^—is complex because people develop bonds throughout their stays, trying to combine the best of the different worlds; and a returnee may feel treated as a ‘foreigner’ in their country of origin.^10^

The complexity of defining (and identifying) return migration challenges not only the study of return migration specifically, but research on migration in general, since migration is often described in terms of transnational practices, and returnees may define themselves as transmigrants. Transnationalism is the process by which migrants build social spaces that connect their country of origin with the country where they are settled, developing and maintaining multiple relationships (family, economic, sociocultural, organisational, religious and political).^12^ These cross-border processes in part occur because migrants take actions, make decisions, feel concerns and develop identities within social spaces that connect several societies simultaneously.^13^

While it is well established that migration is conditioned and conditions individual health risks,^14^ the difficulties noted above have a direct impact on our understanding of the relationship between migration and health. The consideration of return migration within migration and health research has been mainly limited and linked to the empirical generalisation that most immigrants show relatively good health on arrival relative to the host population in their receiving societies (the so-called healthy immigrant paradox).^15–17^ Specifically, return migration has been considered part of the explanation for the mortality advantage through two possible mechanisms. On the one hand, it has been argued that the lack of incentives for returnees to report their emigration to the receiving authorities could be the cause of a mismatch between the numerator and denominator of rates, leading to artificially lower mortality rates compared with the native population in the receiving country.^18–20^ On the other hand, scholars have suggested that sick immigrants might return to their home country seeking care (Salmon bias hypothesis).^21^ However, beyond these hypotheses, which do not always find empirical support,^22 23^ the health of returnees has generally been overlooked in the literature.

Overall, migration research, and especially health-related research, has focused mainly on immigrants in the context of receiving countries, and from a static perspective. Studies on population health address the reality of migration as if it were an event limited in time and, from the cross-sectional point of data collection, but never with a vision of the future or an unfinished stage. Thus, return or intention to return is left out, as well as the factors that determine it, and how return may affect health.

### The role of SDH to understand return migration and returnees’ health

The SDH framework proposed by the WHO in 2010 considers the circumstances in which people are born, grow, live, work and age. Such circumstances include a wide range of factors operating at different levels (from political social and economic forces to family circumstances) shaping people’s health and contributing to health inequities in society.^24^ While the SDH framework has been increasingly employed in health research to identify factors explaining health inequities in receiving countries (typically comparing immigrants vs receiving-country born population), its broader application can also aid in understanding the decision to migrate, stay or return influences health and social inequities on return.

**Figure 2:**
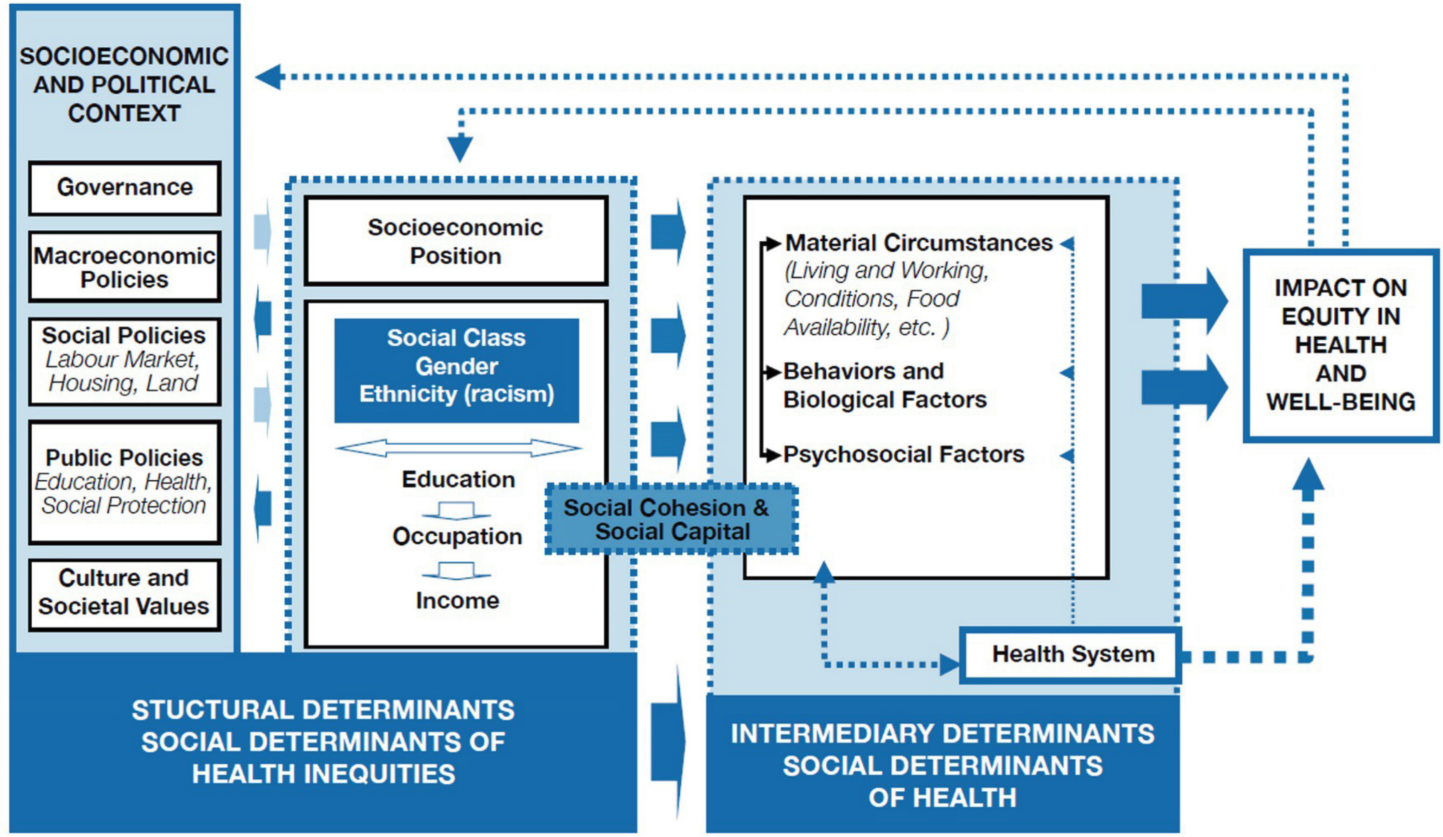
Social determinants of health conceptual framework source: this framework is taken from the WHO.^24^ A conceptual framework for action on the social determinants of health. WHO: https://iris.who.int/handle/10665/44489.^24^

The above framework categorises the SDH into structural, intermediary and a cross-cutting determinants.^24^ Socioeconomic and public policies, governance, culture and social values are seen as structural social determinants, interacting with social stratification factors (social class, gender, ethnicity) to define the socioeconomic position of individuals, which is key in shaping the systems of prestige and discrimination that exist in society and can again influence the sociopolitical context. These structural determinants in turn influence intermediary determinants (material circumstances, behavioural, biological and psychological factors, and health system), which ultimately influence the health of populations, leading to unfair and avoidable inequalities (health inequities). It is also important to note, according to the SDH framework, the role of the cross-cutting determinant ‘social cohesion/social capital’ as a catalyst for health, given that the community (individuals, groups, networks) can have a high level of influence on decision-making and policy development that affect people’s well-being and quality of life. In this context, this cross-cutting determinant signifies elements that influence health outcomes across various social and economic factors, emphasising their interconnected nature within the SDH framework^24^ (figure 2).

Next, we delineate how the structural, intermediary and cross-cutting SDH can influence return migration, consequently influencing social health inequities at both ends.

#### Structural determinants

Socioeconomic and political context factors, such as economic policies (unemployment/end of contract in receiving countries, more jobs/better wages in country of origin, desire to invest savings) and sociocultural values and social and public policies (modernisation of society, rejection of way of life and government policy in origin and receiving countries); and socioeconomic position factors (difficulty of integration in host countries and/or reintegration in countries of origin related to gender, ethnicity or social class inequalities) are all decisive in considering returning.^7 8^ Similarly, the structural conditions of the country of origin are key (eg, poor aid and benefits linked to reintegration policies) to achieve a sustainable return.^10 25–27^

In addition, returnees may also face barriers to reintegration when experiencing social class, ethnic and gender inequalities.^10 26–28^ For example, migrant descendants, who have grown up in societies where they faced ethnic or other inequalities, can experience difficulties integrating in their parents’ or grandparents’ country of origin.^28^ Furthermore, the children who return with their parents can experience marginalisation and educational disadvantage at school (language barriers, differential treatment by teachers), which make integration in their parents’ country of origin difficult and can lead to psychological and other emotional issues.^28^ Migrant women may decide to return to care for their ageing parents, and they may also feel discriminatory treatment on their return; alternatively, they may feel more empowered and valued in their home country because they feel more confident and competent with the capital acquired abroad.^28^

#### Intermediary determinants

Different reasons for returning have been identified: after a successful stay; due to a failed purpose, so migrants had no choice but to return; health problems that prevented them from following through with their plans; homesickness; serious illness or death of a family member that requires their return; or negative experiences in the receiving country.^3 29^

In turn, these reasons also influence the degree of preparedness to return, which may be higher or lower, depending on having achieved the objectives in the receiving country; the opportunities offered by the country of origin; the available resources to undertake the return, such as social networks accumulated and transformed during the migration project, both at destination and at origin (financial and symbolic remittances).^8 30^ A short duration of the migration experience, where the expected benefits (eg, sending remittances, starting a business) are not achieved, may accelerate return.^27^ In addition, the point in the life cycle at which return occurs (marriage, divorce, children’s education, parental ties, health needs and disabilities, etc) is another key factor in preparedness.^8 28^

In relation to the life cycle, age, as biological factor, may contribute significantly to return, as health problems, especially in older people, prevent rather than encourage return,^31 32^ contradicting the ‘salmon bias’.^23 32^ There may also be mixed feelings. A study of migrants residing in Germany, with life-limiting disease, revealed that there was a ‘double home’ experience: access to healthcare and survival were generally worse in the country of origin, but at the same time, there were altered senses of identity, conflicted emotions and ‘longing for home’.^33^ When it comes to the return of children, it is necessary to consider that they are generally less likely to participate in the decision-making of return, their parents deciding on their behalf, and this negatively affects their degree of preparedness.^34^ A high number of returnee children experience psychological impacts (stress-related symptoms, anxiety, depression and anorexia) linked to a lack of sense of belonging (may face ‘double racism’, both in the country they left and in the country they arrived in); conditions which can also affect the process of relocation and adaptation to their parents’ homeland.^34^

Within the psychological factors, Lietaert^35^ points out the importance of using the lens of well-being to discover the complexity and dynamics of post-return situations; for example, when returnees have had better healthcare in the receiving country, they can feel anger and ‘not belonging’ in their country of origin, which can lead to problems of self-esteem and well-being in returnees.^35^ In addition, when migrants have to return (temporarily or permanently) due to caregiving responsibilities for ill parents or to a family bereavement, this is often compounded by a lack of appropriate social and health provisions that can accommodate the needs of families without ‘local’ members who can provide care; this, in turn, can have significant impacts on returnees’ emotional and psychological well-being.^36^

Agency, that is, the capacity to mobilise resources in response to challenges posed by new situations, is also a crucial sociopsychological factor for return decisions, as this can foster a sense of empowerment, driving proactive actions and contributing to transformative changes in the country of origin.^35^ When returnees are motivated and prepared to return, the sustainability of reintegration tends to be greater; specifically, well-planned and voluntary returns have a better chance of successful reintegration.^8^ Those who return often build homes, start businesses, many of them are innovators through a combination of resources (human, financial, cultural and social capital) acquired during their migration experience, and play leadership roles and even get involved in politics.^3 29^

The role of social and health entitlements (Health System), as another powerful intermediary SDH, should also be noted. In many welfare systems, particularly in Europe, these services and benefits have generally been linked to residence or employment status and social vulnerability (eg, disability, low income), excluding undocumented migrants and those working in the informal economy.^37^ Even if entitled, migrants often face barriers in accessing these services and benefits due to discrimination and a lack of knowledge in navigating the system.^37–39^ Many migrants have to seek strategies, drawing on formal and informal resources, to be able to access the services and benefits of the Welfare State, which has been called Transnational Bricolage of Health protection.^40^ However, this is often still precarious because many of the tactics used are pushing the limits of policy regulations in origin or destination countries or transgressing them.^39^ In the European Union/European Economic Area (EU/EEA) context, for example, these practices include not registering at the consulate so as not to lose their European Health Insurance Card rights, as well as their right to access the national health services in origin; or requesting a certificate of registration at the parents' home address to reactivate the health card when it has been deactivated.

There is a strong evidence of the health inequities suffered by the immigrant population, which together with the lack of sociofamilial support in the receiving country, as well as the lack of guarantees of access to quality health services,^14 41^ can become triggers for return (eg, older Britons returning to the UK from Spain with high levels of isolation, mental illness, alcoholism, increasing dependency and bodily deterioration^42^, and who are likely to return because they are frail and in need of care^43^). Furthermore, older people and/or people with health problems experience more insecurity in the degree of preparedness, as they need to ensure that they will have access to health and social services on their return.^44^

#### Cross-cutting determinant

Finally, the cross-cutting social determinant ‘social cohesion/social capital’ plays a crucial role in facilitating return. Having family ties and social networks of attachment, as well as feelings of welcome in the country of origin, provide a foundation of control and security by making it possible to reserve a place ‘at home’ for migrants who wish or need to return.^8 25 45^ Indeed, children of returnees, who were born and have grown up in the migrants’ parents receiving country, may have a lack identification with the parents’ country of origin.^34^

In addition, social remittances—defined as the ideas, behaviours, identities and social capital that flow from communities in receiving countries to those in sending countries^46^—are an important coping mechanism for the uncertainty associated with return, as they play a relevant role in migrant entrepreneurship, community, family formation and political integration.^25^ Vertovec^47^ sees social remittances as a premise of transnationalism that generates not only financial but also social capital, given that there is a dissemination of assets when at least one of the contexts (receiving country or country of origin) is unstable for political reasons, racism, bureaucracy or difficult labour market conditions. Social remittances, which are thus constituted as social capital, not only transform societies in the countries of origin but also become facilitators of return.

## Concluding remarks

In this paper, we have sought to analyse on the implications of the SDH in the decision of return, considering that migration movements are neither stable nor definitive, but just another stage in the migration circle.

The SDH framework can help researchers to better understand the phenomenon of return migration and, therefore, to have a more comprehensive understanding of the health of the migrant population. It can also be instrumental in designing future studies on the health of returnees, and thus contribute to the design of policies aimed at facilitating return and promoting their health. Furthermore, it encourages interdisciplinary collaboration to investigate how social factors are embodied, giving rise to health inequities that are intricately linked to the migration experience.

## Data Availability

There are no data in this work.
